# IMPLEMENTING THE 4MS IN PRIMARY CARE: BUILDING AN AGE-FRIENDLY HEALTH SYSTEM

**DOI:** 10.1093/geroni/igz038.2828

**Published:** 2019-11-08

**Authors:** Ellen Flaherty, Terry Fulmer

**Affiliations:** 1 Dartmouth-Hitchcock Medical Center, Lebanon, New Hampshire, United States; 2 The John A. Hartford Foundation, New York, New York, United States

## Abstract

The Age Friendly Health Systems initiative is a culture change movement funded by the John A. Hartford Foundation in collaboration with the Institute for Health Care Improvement. Transforming clinical training environments into integrated geriatrics and primary care systems to become Age-Friendly Health Systems must incorporate the principles of value-based care and alternative-payment models. This symposium will discuss how the implementation of the Geriatric Interprofessional Team Transformation in Primary Care (GITT-PC) model and the Reducing Avoidable Facility Transfer Model (RAFT) in primary care will improve patient outcomes focused on the 4M’s of the Age Friendly Health System. The success of the GITT-PC model focuses on 4 Medicare reimbursable services including the Annual Wellness Visit, Transitional Care Management, Chronic Care Management and Advance Care Planning. The RAFT model focuses on What Matters Most to residents of long term care facilities and reduces ED visits and hospital transfers through elicitation of goals of care and 24 hour virtual support from an interprofessional geriatric team.

